# Mortality after radiotherapy or surgery in the treatment of early-stage non-small-cell lung cancer: a population-based data analysis in the clinical cancer registry of Brandenburg-Berlin

**DOI:** 10.1007/s00066-023-02055-z

**Published:** 2023-03-13

**Authors:** Jörg Andreas Müller, Dirk Vordermark, Daniel Medenwald

**Affiliations:** 1 Department of Radiation Oncology, University Hospital Halle (Saale), Ernst-Grube-Str. 40, 06120 Halle (Saale), Germany; 2 Institute of Medical Epidemiology, Biometry, and Informatics, Martin Luther University Halle-Wittenberg, Magdeburger Straße 8, 06112 Halle (Saale), Germany

**Keywords:** NSCLC, Early-stage lung cancer, SBRT, UICC stage I, UICC stage II, Lung cancer

## Abstract

**Purpose:**

Stereotactic body radiotherapy (SBRT) is an established treatment method with favorable toxicity for inoperable early-stage non-small-cell lung cancer (NSCLC) patients. This paper aims to evaluate the importance of SBRT in the treatment of early-stage lung cancer patients compared to surgery as standard of care.

**Methods:**

The German clinical cancer register of Berlin-Brandenburg was assessed. Cases of lung cancer were considered if they had a TNM stage (clinical or pathological) of T1-T2a and N0/x and M0/x, corresponding to UICC stages I and II. In our analyses, cases diagnosed between 2000 and 2015 were included. We adjusted our models with propensity score matching. We compared patients treated with SBRT or surgery regarding age, Karnofsky performance status (KPS), sex, histological grade, and TNM classification. Further, we assessed the association of cancer-related parameters with mortality; hazard ratios (HR) from Cox proportional hazards models were computed.

**Results:**

A total of 558 patients with UICC stages I and II NSCLC were analyzed. In univariate survival models, we found similar survival rates in patients who underwent radiotherapy compared with surgery (HR 1.2, 95% confidence interval [CI] 0.92–1.56; *p* = 0.2). Our univariate subgroup analyses of patients > 75 years showed a statistically nonsignificant survival benefit for patients treated with SBRT (HR 0.86, 95% CI 0.54–1.35; *p* = 0.5). Likewise, in our T1 subanalysis, survival rates were similar between the two treatment groups regarding overall survival (HR 1.12, 95% CI 0.57–2.19; *p* = 0.7). The availability of histological data might be slightly beneficial in terms of survival (HR 0.89, 95% CI 0.68–1.15; *p* = 0.4). This effect was also not significant. Regarding the availability of histological status in our subgroup analyses of elderly patients, we could show similar survival rates as well (HR 0.70, 95% CI 0.44–1.23; *p* = 0.14). T1-staged patients also had a statistically nonsignificant survival benefit if histological grading was available (HR 0.75, 95% CI 0.39–1.44; *p* = 0.4). Concerning adjusted covariates, better KPS scores were associated with better survival in our matched univariate Cox regression models. Further, higher histological grades and TNM stages were related to a higher mortality risk.

**Conclusion:**

Using population-based data, we observed an almost equal survival of patients treated with SBRT compared to surgery in stage I and II lung cancer. The availability of histological status might not be decisive in treatment planning. SBRT is comparable to surgery in terms of survival.

**Supplementary Information:**

The online version of this article (10.1007/s00066-023-02055-z) contains supplementary material, which is available to authorized users.

## Introduction

Lung cancer accounts for a high proportion of cancer mortality [1–3]. Improvements in overall survival have remained small over the past decades [4], with differing treatment strategies across the world.

For early-stage (UICC I) non-small-cell lung cancer (NSCLC), surgery is the standard of care, with video-assisted thoracoscopic (VATS) lobectomy as the most common therapeutic approach.

In fact, a mentionable proportion of early-stage NSCLC patients cannot be treated surgically due to tumor location, operative risk, age, frailty, or comorbidities [5–8]. Before the introduction of stereotactic body radiation therapy (SBRT), conventional radiotherapy was the only practicable treatment option for inoperable patients, with a minor improvement in survival compared to untreated patients [6, 7].

SBRT is a high-precision image-guided form of radiotherapy (RT) characterized by the application of few fractions of high biologic effective doses to small tumor volumes [9]. With the introduction of SBRT, tumor control and outcome could be significantly improved [10–12]. Furthermore, local tumor control rates after SBRT are reported with limited toxicity in over 90% [13], which indicates that this treatment is a suitable option for elderly patients and those with relevant comorbidities.

The impact of new treatment methods can be effectively assessed by population-based studies [10]. Haasbeek et al. [14] showed an improvement in survival of elderly early-stage NSCLC patients treated with SBRT in a population-based study in the Netherlands. Similarly, Palma et al. [10] showed a 16% absolute increase in RT use, improved overall survival (OS), and a reduction in untreated patients after stereotactic ablative radiotherapy (SABR) was introduced for NSCLC stage I patients aged 75+ in the Netherlands. Likewise, a study by Ostheimer et al. in German cancer registry data observed a survival improvement in stage I lung cancer over time.

With increasing use of radiotherapy, a greater improvement occurred in patients treated with radiotherapy as compared to surgery [15].

Although German radiation oncology centers rapidly adopted SBRT as an alternative treatment for surgery after the year 2000 [13], there are only few population-based analyses on the impact of the introduction of SBRT in Germany available so far. The aim of this study is to evaluate changes in OS associated with radiotherapy compared to surgery on an aggregated level for early-stage NSCLC patients (stage I–IIa) based on the general population of lung cancer patients.

## Methods

### Data and material

For our analyses, we used data provided by the clinical cancer registry of Berlin-Brandenburg for public use. This population-based registry is regulated by German federal law and incorporates data that are transferred from hospitals in Berlin or Brandenburg.

The dataset contains, amongst other information, data on TNM stage, grading and histology, date of birth, cause and date of death, date of diagnosis (month as smallest temporal unit in each date variable), and treatment. Further, information on treatment procedures such as administered radiation dose and fractionation or number of surgeries are included. In addition, the TNM stage refers in this dataset to the clinical or pathological stage (if an operation was performed).

**Fig. 1:**
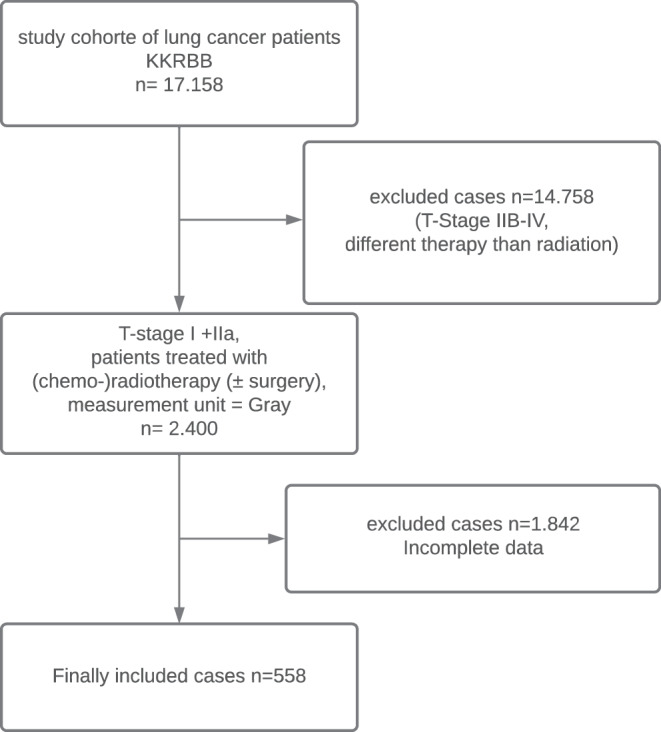
Flowchart of sample inclusion and exclusion criteria

We considered cases of lung cancer for our analyses if they had a TNM stage (clinical or pathological) of T1-T2a and N0/x and M0/x, corresponding to the UICC stages I and II. Furthermore, we excluded all cases with a small-cell histology. Likewise, cases were excluded if there were no or inconsistent records of radiotherapy and if analyzed data had missing values. Finally, 558 cases were considered for further analyses (Fig. 1).

### Definition of periods

In a survey including nearly all radiotherapy institutions in Germany [13], the application of SBRT increased continuously between 1998 and 2011, while the cumulative numbers tripled in that period. In our analyses, cases diagnosed between 2000 and 2015 (most recent data with sufficient quality) were included. Cases were censored on December 2016 (latest complete recording of death) or after 60 months, to avoid bias due to cases that died in more recent years but whose changed survival status had not yet been considered in the data.

### Grade definition

Grade categories were defined as low grade (grades 1, 2, and low/medium grade; definition according to the *Arbeitsgemeinschaft Deutscher Tumorzentren* [ADT]) and high grade (grades 3, 4, and high grade, ADT definition [16]).

### Statistical analyses

We used Cox proportional hazard regression models to assess the association of cancer-related parameters with mortality and computed hazard ratios (HR) with 95% confidence intervals (CI).

To reduce selection bias, we adjusted our models with propensity score matching. Propensity score matching is a statistical technique in which a treatment case is matched with one or more control cases based on each measured propensity score. This matching can help strengthen causal arguments in observational studies [17]. Thus, all cancer-related parameters were matched for cases treated with surgery or SBRT before Cox regression analyses were performed.

Furthermore, we computed univariate and multivariate Cox regression models. All models were adjusted for histological grade (as defined above), age, T stage (T1a vs. T1b vs. T2a; if the subclassification of T2 was not available in the data, we declared these cases as subgroup T2), Karnofsky performance status (KPS), patient sex, and SBRT without surgery. In addition, a subgroup analysis was performed for patients older than 70 years to investigate a possible benefit of SBRT over surgery in elderly patients. In all treatment groups, histological confirmation was unavailable for all cases. To assess potential bias due to missing data, we computed Kaplan–Meier survival curves and Cox regression models for patients with known histological status and for patients without histological grading. All analyses were also performed for T1-staged patients.

During the studied period (2000–2014), the change from TNM version 5 to 6 (since 2002) did not change the TNM stage of considered patients (only T1 was included). The following TNM version 7 (since 2009) introduced the new levels of T1a and T1b and, additionally, T2a, while patients with the latter stage might also be candidates for SBRT.

In addition, we analyzed the quality of the data by focusing on missing data in cases of elderly patients. We examined the structure and quantity of missing values using the MICE package (multivariate imputation by chained equations) of the statistical software RStudio (RStudio 2020, Integrated Development for R. RStudio, PBC, Boston, MA, USA). We assumed that there might be a higher percentage of missing values in older patients, leading to an age-related bias.

Moreover, the inclusion of only one cancer registry might lead to a selection bias, although the cancer registry of Berlin-Brandenburg distinguishes itself from other registries by a higher data quality and longer recording time. We chose the cancer registry of Berlin-Brandenburg from all German federal states because of continuous registration activity during the study period (2000–2015) and the availability of Eastern Cooperative Oncology Group (ECOG)-related Karnofsky performance status scale (KPS) data.

A significance level of 5% was used. All statistical analyses were performed using RStudio version 1.4.1717.

## Results

### Case selection

The total number of UICC stage I–VI lung cancer cases diagnosed between 2000 and 2015, as provided by the cancer registry of Berlin-Brandeburg, was 17,158 (cases were reported in accordance with international guidelines, see http://www.iacr.com.fr/). In the next step, cases treated without radiation therapy or with T stage IIB-VI were removed. Finally, we excluded cases with missing treatment variables, which resulted in a final number of 558 cases that we used for univariate and multivariate analyses (Fig. 1).

### Patient characteristics

Regarding the distribution of considered treatments, we found a higher proportion of cases treated only by surgery (73.9%) when compared with SBRT (26.2%). In relative terms, patients treated with radiotherapy were noticeably younger when compared to proportions in the surgery group (*p* < 0.001). Despite the difference in age, there was no mentionable difference in sex (*p* = 0.3). Furthermore, there was no significant difference in the proportion of histological grades in either group (*p* = 0.5). In both treatment groups, histological confirmation was unavailable for all cases (missing values SBRT: *n* = 105; surgery: *n* = 68). The comparison of KPS levels revealed a significant difference between SBRT and surgery cases (*p* = 0.002). Patients treated with SBRT were more likely to have a better performance status than surgical patients. The proportion of cases receiving radiotherapy in clinical stage T1a or T1b was significantly lower than the proportion receiving surgery (*p* < 0.001; Table 1). For the SBRT group without surgery, median survival was 19 months (95% CI 14–26 months), and in the subgroup > 75 years, median survival was 27 months (95% CI 19–50 months). In contrast, for patients treated with surgery, median survival was 22 months (95% CI 20–26 months), and in the subgroup > 75 years, median survival was 24 months (95% CI 18–30 months).

**Table 1:** Patient characteristics in the study cohort

Characteristic	*N*	Stereotactic body radiotherapy vs. surgery		*p*-value^b^
		SBRT^a^	Surgery^a^	
*Sex*	558	–	–	0.3
Male	–	315/412 (76%)	105/146 (72%)	–
Female	–	97/412 (24%)	41/146 (28%)	–
*Patient age*	558	64.48 (9.81)	70.18 (10.27)	< 0.001
*Karnofsky performance status*	556	–	–	0.002
30–40%	–	25/411 (6.1%)	15/145 (10%)	–
50–60%	–	154/411 (37%)	70/145 (48%)	–
70–80%	–	157/411 (38%)	50/145 (34%)	–
90–100%	–	75/411 (18%)	10/145 (6.9%)	–
Unknown	–	1	1	–
*Histological grade*	385	–	–	0.5
G1	–	11/307 (3.6%)	1/78 (1.3%)	–
G2	–	126/307 (41%)	32/78 (41%)	–
G3	–	159/307 (52%)	40/78 (51%)	–
G4	–	11/307 (3.6%)	5/78 (6.4%)	–
Unknown	–	105	68	–
*TNM classification*	558	–	–	< 0.001
1a	–	18/412 (4.4%)	18/146 (12%)	–
1b	–	24/412 (5.8%)	29/146 (20%)	–
2	–	286/412 (69%)	70/146 (48%)	–
2a	–	84/412 (20%)	29/146 (20%)	–

*SBRT* stereotactic body radiotherapy

^a^*n*/*N* (%); mean (standard deviation)

^b^Pearson’s chi-squared test, Wilcoxon rank sum test, Fisher’s exact test

### Propensity score matching

We used propensity score analyses to adjust our models to reduce selection bias. All included RT cases were matched with one control case based on each measured propensity score. We used the nearest-neighbor method to perform our analyses. Overall, a sample size of *n* = 292 cases was included; 78 cases could be matched in the control and treatment groups. SBRT was defined as the treatment variable, surgery was stated as the control variable.

In short, the treated and control SBRT vs. surgery cases after matching were very similar in terms of age, KPS, sex, histological grade, and TNM classification (Table S1 of the supplement shows the numeric results of propensity score matching).

**Fig. 2:**
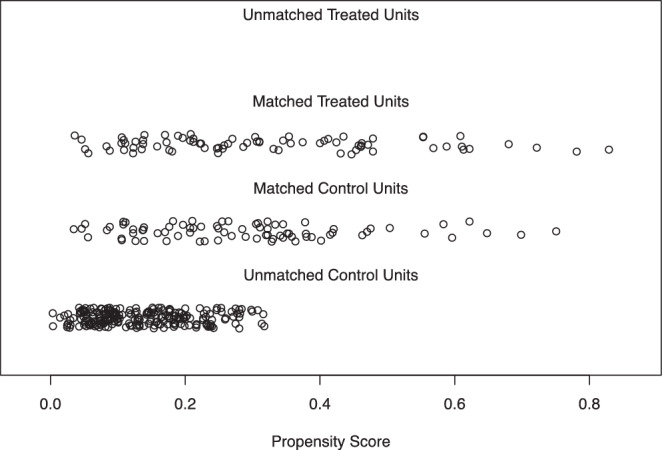
Distribution of propensity scores

Figure 2 is a jitter plot where each case represents a case’s propensity score. The absence of cases in the uppermost stratification indicates that there were no unmatched treatment units. The middle stratifications show the close match between the treatment units and the matched control units. The final stratification shows the unmatched control units, which will not be used in any further analyses.

Figure S1 shows the histograms before and after matching.

Furthermore, we calculated a patient characteristics table for matched cases (Table 2). This table shows that most of the matched patients were male and there was no significant difference in terms of age between the matched SBRT and surgery group (*p* = 0.1). The adjustment of SBRT and surgery groups regarding the analyzed characteristics of histological grading and KPS shows almost equal data. Almost half of the matched patients had a KPS of 50–60% and a histological G3 grading. In 69 cases for the SBRT group and 68 cases for the surgery group, histological grades were unknown. Only the TNM classification differed significantly between the two groups (*p* = 0.006).

**Table 2:** Patient characteristics of matched data

Characteristic	*N*	Stereotactic body radiotherapy vs. Surgery		*p*-value^b^
		SBRT^a^	Surgery^a^	
*Sex*	292	–	–	> 0.9
Male	–	105/146 (72%)	105/146 (72%)	–
Female	–	41/146 (28%)	41/146 (28%)	–
*Patient age*	292	68.59 (8.88)	70.18 (10.27)	0.10
*Karnofsky performance status*	290	–	–	0.3
30–40%	–	16/145 (11%)	15/145 (10%)	–
50–60%	–	75/145 (52%)	70/145 (48%)	–
70–80%	–	37/145 (26%)	50/145 (34%)	–
90–100%	–	17/145 (12%)	10/145 (6.9%)	–
Unknown	–	1	1	–
*Histological grade*	155	–	–	0.5
G1	–	0/77 (0%)	1/78 (1.3%)	–
G2	–	30/77 (39%)	32/78 (41%)	–
G3	–	45/77 (58%)	40/78 (51%)	–
G4	–	2/77 (2.6%)	5/78 (6.4%)	–
Unknown	–	69	68	–
*TNM classification*	292	–	–	0.006
1a	–	14/146 (9.6%)	18/146 (12%)	–
1b	–	15/146 (10%)	29/146 (20%)	–
2	–	99/146 (68%)	70/146 (48%)	–
2a	–	18/146 (12%)	29/146 (20%)	–

*SBRT* stereotactic body radiotherapy

^a^*n*/*N* (%); mean (standard deviation)

^b^Pearson’s chi-squared test, Wilcoxon rank sum test, Fisher’s exact test

### Survival analyses

We computed univariate Cox regression models for the matched data and analyzed patients older than 75 years separately. As we started the analyses, we noted a lot of missing histological data. Thus, we decided to add the availability of the histological status as a parameter to our regression model.

In univariate survival models, we could not find better survival in patients who underwent radiotherapy compared with surgery (HR 1.2, 95% CI 0.92–1.56; *p* = 0.2); while the null effect value was included in the confidence interval for these cases, the advantage for surgery was not statistically significant. Likewise, in our T1 subanalysis, survival rates between the two treatment groups were similar in terms of survival (HR 1.12, 95% CI 0.57–2.19; *p* = 0.7; Table S2). Our univariate subgroup analyses of patients > 75 years showed a statistically nonsignificant benefit in terms of survival for patients treated with SBRT (HR 0.86, 95% CI 0.54–1.35; *p* = 0.5).

**Fig. 3:**
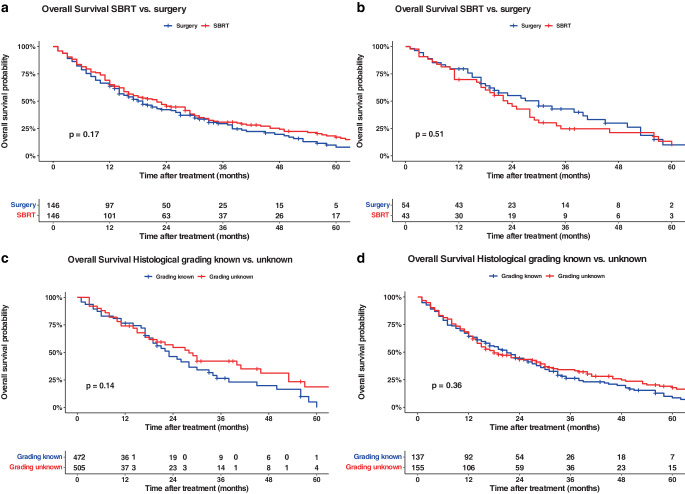
Kaplan–Meier curves of all patients (*curves* **a**, **c**) and patients > 75 years (*curves* **b**, **d**) data (outcome: overall survival). *Curves* **a**, **b** show the analyses based on patients treated with surgery (arm A) vs. patients treated with SBRT (arm B). The *curves* **c**, **d** are based on the availability of histological grades (arm A) vs. unknown histological status (arm B). All graphs were censored 60 months after treatment

**Fig. 4:**
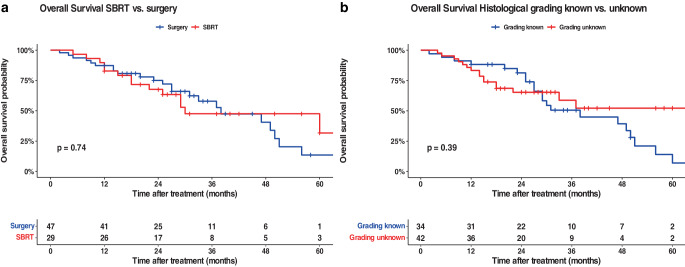
Kaplan–Meier curves of T1-staged patients (outcome: overall survival): **a** shows the analyses based on patients treated with surgery (arm A) vs. patients treated with SBRT (arm B); **b** is based on the availability of histological grades (arm A) vs. unknown histological status (arm B). All graphs were censored 60 months after treatment

The availability of histological data might be slightly beneficial in terms of survival. This effect was also nonsignificant (HR 0.89, 95% CI 0.68–1.15; *p* = 0.4). T1-staged patients also had a statistically nonsignificant survival benefit if histological grading was available (HR 0.75, 95% CI 0.39–1.44; *p* = 0.4; Table S2). In our subgroup analyses of elderly patients, however, we could show a statistically nonsignificantly prolonged survival rate as well (HR 0.70, 95% CI 0.44–1.23; *p* = 0.14). In our data, male patients had a higher mortality risk than women, although this was not significant (HR 1.18, 95% CI 0.88–1.58; *p* = 0.3 for all patients; HR 1.26, 95% CI 0.76–2.07; *p* = 0.4 for patients > 75 years). Regarding adjusted covariates, better KPS scores were associated with better survival in our matched univariate Cox regression models. Further, higher histological grades and TNM stages were related to a higher mortality risk. Caused by the change in TNM classification during the study period, some patients who would have been classified as stage T2b in the new classification system might be included in the stage 2 category, leading to a higher mortality risk compared to the T2a group (see Tables 3, 4). Survival data are shown in Fig. 3 and 4.

**Table 3:** Univariate Cox regression model of propensity-matched data

Characteristic	*N*	HR	95% CI	*p*-value
*Sex*	558			0.3
Female		—	—	
Male		1.18	0.88, 1.58	
*Patient age*	292	0.99	0.98, 1.01	0.2
*Karnofsky performance status*	290			< 0.001
30–40%		—	—	
50–60%		0.97	0.64, 1.47	
70–80%		0.72	0.46, 1.14	
90–100%		0.34	0.18, 0.64	
*Histological grade*	155			0.067
G1		—	—	
G2		2.16	0.29, 15.9	
G3		3.28	0.44, 24.3	
G4		4.58	0.53, 39.3	
*TNM classification*	292			< 0.001
1a		—	—	
1b		1.15	0.59, 2.24	
2		2.64	1.53, 4.57	
2a		2.28	1.22, 4.24	
*Histological grading available*	292	0.89	0.68, 1.15	0.4
*Treated with SBRT*	292	1.20	0.92, 1.56	0.2

*HR* hazard ratio, *CI* confidence interval, *SBRT* stereotactic body radiotherapy

**Table 4:** Univariate Cox regression model of propensity-matched data for patients > 75 years

Characteristic	*N*	HR	95% CI	*p*-value
*Sex*	97	N/A	N/A	0.4
Female	N/A	—	—	N/A
Male	N/A	1.26	0.76, 2.07	N/A
*Patient age*	97	1.02	0.94, 1.10	0.6
*Karnofsky performance status*	97	N/A	N/A	0.004
30–40%	N/A	—	—	N/A
50–60%	N/A	0.66	0.33, 1.33	N/A
70–80%	N/A	0.35	0.15, 0.78	N/A
90–100%	N/A	0.24	0.08, 0.66	N/A
*Histological grade*	50	N/A	N/A	0.12
G1	N/A	—	—	N/A
G2	N/A	2.86	0.35, 23.6	N/A
G3	N/A	3.35	0.42, 27.0	N/A
G4	N/A	14.3	1.28, 159	N/A
*TNM classification*	97	N/A	N/A	< 0.001
1a	N/A	—	—	N/A
1b	N/A	0.94	0.29, 3.00	N/A
2	N/A	1.71	0.61, 4.81	N/A
2a	N/A	1.90	0.63, 5.76	N/A
*Histological grading available*	97	0.70	0.44, 1.12	0.14
*Treated with SBRT*	97	0.86	0.54, 1.35	0.5

*HR* hazard ratio, *CI* confidence interval, *SBRT* stereotactic body radiotherapy

### Missing values analyses

We assumed that there might be a higher percentage of missing values in older patients, leading to an age-related bias. Therefore, we computed a missing value analysis comparing all patients with the subgroup of patients > 75 years regarding the documented SBRT radiation dose and performed surgeries. The cancer registry collected single-fraction and cumulative radiation doses. We assumed based on this information that three-fraction SBRT was used if single-fraction radiation doses > 4 Gy were documented.

Regarding surgery, OPS (*Operationen- und Prozeduren-Schlüssel*) codes for each procedure were documented. Thoracic surgeons performed lobectomies in all documented cases. If there were inconsistencies in the documentation of the performed surgeries, e.g., missing date or OPS code, we defined the case as a missing value.

SBRT radiation doses of all included patients were missing in 37.81% of cases. The proportion of missings for the second radiation was 69.53% (third RT dose: 86.37%; fourth RT dose 94.98%). For the elderly patients, 26.82% of the first RT doses were missing. The second dose was missing in 71.54% of cases (third RT dose: 90.24%; fourth RT dose 96.74%).

**Fig. 5:**
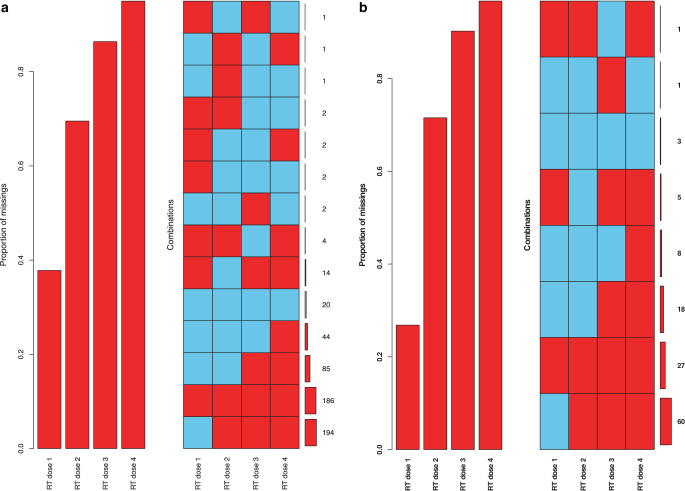
Proportion and combination of missing values for all patients (**a**) and patients > 75 years (**b**). This figure shows missing values in the documented SBRT radiation doses of the first four radiations. The combination matrix shows all variations of missings (*red*) and values (*blue*) of the first four SBRT doses. Overall, up to 13 radiation doses were collected by the cancer registry of Berlin-Brandenburg. Only a small number of patients were documented with > 5 irradiations, so we decided to exclude them for clarity of presentation

Moreover, the percentage of missing values in the first performed surgery was 26.16% for all included patients (second surgery: 58.78%; third surgery: 77.59%; fourth surgery: 86.37%). In contrast to this, the percentage of missings for elderly patients was 43.9% for the first operation (second surgery: 73.17%; third surgery: 87.80%; fourth surgery: 91.86%). Inconsistent combinations of documented radiation doses were excluded from further analysis. Information on missings is provided in Fig. 5.

## Discussion

In our study, the proportion of cases treated with radiotherapy was smaller when compared to surgery. To address this selection bias, we used propensity score matching. This method allowed us to create two balanced treatment groups with identical sample sizes (*n* = 78).

In our Cox regression models, patients age had no significant influence on survival. Nonetheless, we created a subgroup of patients older than 75 years because of prior studies showing a lower risk of death in the time period with wide availability of SBRT (after 2007) compared to the time between 2000 and 2003 with limited availability of this treatment for this particular group [15]. Furthermore, Palma et al. [10] showed improved OS and a reduction in untreated patients after SBRT was introduced for NSCLC stage I patients aged 75+ in the Netherlands. According to these findings, we had better survival in the SBRT group compared to the surgery group of elderly patients. This effect was statistically nonsignificant due to the limited number of cases.

In fact, the majority of patients had a slightly better outcome when they were treated by surgery. This benefit was not statistically significant considering our survival analyses. When compared to surgery, the survival improvement in cases treated with radiotherapy was greater in elderly patients.

The surgery group showed a lower mortality risk compared to the SBRT group, which might be biased, as this group in general is highly selective in terms of better performance status and fewer comorbidities. In our study, however, patients treated by surgery had worse KPS scores. Such a disadvantageous survival prospect might become apparent when stereotactic radiotherapy is a widely available treatment option for patients in a poor health condition. Population-based survival data of German lung cancer patients show a clear dependence on stage, where UICC stage Ia patients have a 5-year survival rate of 64.9/73.9% (male/female) and stage Ib patients of 53.1/64.2% (male/female) [18]. We could show a clear dependence on UICC stage in terms of survival in our analyses as well. Stage II patients had higher hazard ratios compared to stage I patients.

Regarding the minor sex difference in our study, a worse survival prospect and health status in men compared to women might make them the main beneficiaries of emerging technologies for patients who are unfit for surgery or conventional radiotherapy. Furthermore, the lower mortality risk of women found in our study corresponds with the general population-based survival rates of German lung cancer patients, where male patients have significantly inferior survival compared to female patients [18].

In our dataset, we did not consider survival in relation to surgery or radiotherapy compared to the period before SBRT was widely available. In this context, Haasbeek et al. [14] found improved survival in the subgroup of patients treated with radiotherapy or surgery in the Netherlands. Accordingly, analysis of German cancer registry data showed the same improvement in survival for both treatment methods [15]. In contrast, Palma et al. reported a survival improvement confined to the radiation group. In the analysis by Haasbeck et al., there was no change in survival of the untreated group [10, 14]. A German study on diagnosis-related group statistics suggested that radiotherapy remains a discipline with an important inpatient component [19]. In sum, further retrospective analyses in this field should address differing periods of SBRT availability in Germany.

Improvements in surgical techniques and increased use of diagnostic imaging such as fluorodeoxyglucose positron-emission tomography (FDG-PET) may have also affected both treatment groups. The strongest impact of FDG-PET on treatment-related mortality might result from stage migration to higher stages due to PET imaging [20, 21]. FDG-PET diagnostics might move subjects with an adverse survival prospect (high FDG uptake) to higher stages and bias survival analyses towards a virtual improvement. Additionally, better compliance with cancer treatment might have an influence on better survival in the most recent period [22].

Sun et al. analyzed 5018 hospital- and 712 population-based NSCLC cases of all stages regarding the prognostic value of histological grades. Their findings showed a significant prognostic value for survival in NSCLC patients. Hence, they recommended considering histological grading as an independent parameter in treatment planning beyond TNM staging [23]. However, in our findings, we have seen a slight, statistically nonsignificant survival benefit for all patients treated with SBRT and the subgroup of elderly patients. Based on our data, it might not always be necessary to base therapeutic decisions on the availability of histological grading, especially if the immunohistochemical examination would lead to a delayed start of therapy. Further research based on a more extensive database is needed to achieve a clearer picture on the predictive value of histological grading in early-stage NSCLC.

For elderly patients in Germany, significantly reduced survival was previously reported in patients older than 80 years compared to patients younger than 60 years (8.4% vs. 18.5% for 5 years) [18]. In contrast, in our data, we found better median survival rates for elderly patients in both treatment groups. This effect could be explained by differing comorbidities. Thus, there might be a selection bias in the group of patients > 75 years due to allocating fitter patients to both the treatment groups. Unfortunately, information on severe comorbidities is limited in registry data. Therefore, it would be useful to include additional clinical parameters for future analysis.

In terms of missing values, there was a higher percentage of missing RT doses and performed surgeries in the subgroup of elderly patients, leading to a presumed age-related bias. Concerning this matter, it might be possible that due to age-related comorbidities, fewer surgeries were performed in the subgroup of elderly patients. Our missing values matrix, however, showed consistently documented surgeries for patients > 75 years. Likewise, only a small number of cases had inconsistently documented SBRT doses. Thus, the differences between the two groups might not be primarily age related. Future analysis should address age-related differences in data quality, especially in public health research.

## Conclusion

By considering population-based data, we found almost equal survival in patients treated with SBRT compared to surgery in stage I and II lung cancer. The availability of histological status might not be decisive for treatment planning, based on our findings. To our knowledge, this study is one of the first German approaches to analyze mortality in early-stage NSCLC patients treated with SBRT or surgery. Future research in this field should also include more cancer registries to reach a more extensive database. Furthermore, adding clinical information to the existing registry data would allow for more profound analysis. From a public health perspective, SBRT is a good therapeutic option in terms of survival, especially for elderly and inoperable patients.

## Supplementary Information

The supplement provides information on numeric results and figures of propensity score matching, as well as propensity score matched univariate cox regression models for T1‐staged patients.

## Abbreviations

CI: Confidence interval
ECOG: Eastern Cooperative Oncology Group
FDG-PET: Fluorodeoxyglucose positron-emission tomography
HR: Hazard ratio
KPS: Karnofsky performance status scale
NSCLC: Non-small-cell lung cancer
OS: Overall survival
RT: Radiotherapy
SABR: Stereotactic ablative radiotherapy
SBRT: Stereotactic body radiotherapy
SCLC: Small-cell lung cancer
UICC: Union for International Cancer Control
VATS: Video-assisted thoracoscopic surgery
