# Supplementary material for: Mortality after radiotherapy or surgery in the treatment of early-stage non-small-cell lung cancer: a population-based data analysis in the clinical cancer registry of Brandenburg-Berlin
Source: Strahlenther Onkol. 2023 Mar 13;199(7):658–67. doi: 10.1007/s00066-023-02055-z (PMC10281903; doi:10.1007/s00066-023-02055-z)
Supplement: Supplementary file 1 — The supplement provides information on numeric results and figures of propensity score matching, as well as propensity score matched univariate cox regression models for T1‐staged patients. [file 66_2023_2055_MOESM1_ESM.docx]

# Supplement

Propensity Score Matching SBRT vs. surgery

Summary of Balance for All Data:

Means Treated Means Control Std. Mean Diff. Var. Ratio

distance 0.3165 0.1742 0.7553 2.3624

Age 69.6923 64.1013 0.5650 1.0028

Sex

Male 0.7692 0.7843 -0.0358 .

Female 0.2308 0.2157 0.0358 .

Karnofsky Perfomance Status

30-40% 0.1026 0.0490 0.1765 .

50-60% 0.4744 0.3595 0.2301 .

70-80% 0.3205 0.3954 -0.1605 .

90-100% 0.1026 0.1961 -0.3082 .

Histological Grade

G1 0.0128 0.0359 -0.2056 .

G2 0.4103 0.4085 0.0036 .

G3 0.5128 0.5196 -0.0136 .

G4 0.0641 0.0359 0.1149 .

TNM-Classification

T1a 0.1538 0.0425 0.3087 .

T1b 0.1538 0.0556 0.2724 .

T2 0.5128 0.6961 -0.3666 .

T2a 0.1795 0.2059 -0.0688 .

eCDF Mean eCDF Max

distance 0.2331 0.3788

Age 0.1130 0.2360

Sex

Male 0.0151 0.0151

Female 0.0151 0.0151

Karnofsky Perfomance Status

30-40% 0.0535 0.0535

50-60% 0.1149 0.1149

70-80% 0.0749 0.0749

90-100% 0.0935 0.0935

Histological Grade

G1 0.0231 0.0231

G2 0.0018 0.0018

G3 0.0068 0.0068

G4 0.0282 0.0282

TNM-Classification

T1a 0.1114 0.1114

T1b 0.0983 0.0983

T2 0.1833 0.1833

T2a 0.0264 0.0264

Summary of Balance for Matched Data:

Means Treated Means Control Std. Mean Diff. Var. Ratio

distance 0.3165 0.2879 0.1518 1.4084

Age 69.6923 68.9487 0.0751 1.2957

Sex

Male 0.7692 0.7692 0.0000 .

Female 0.2308 0.2308 0.0000 .

Karnofsky Performance Status

30-40% 0.1026 0.1154 -0.0423 .

50-60% 0.4744 0.4872 -0.0257 .

70-80% 0.3205 0.2692 0.1099 .

90-100% 0.1026 0.1282 -0.0845 .

Histological Grade

G1 0.0128 0.0128 0.0000 .

G2 0.4103 0.3462 0.1303 .

G3 0.5128 0.5769 -0.1282 .

G4 0.0641 0.0641 0.0000 .

TNM-Classification

T1a 0.1538 0.1154 0.1066 .

T1b 0.1538 0.1410 0.0355 .

T2 0.5128 0.5641 -0.1026 .

T2a 0.1795 0.1795 0.0000 .

eCDF Mean eCDF Max Std. Pair Dist.

distance 0.0165 0.1667 0.1531

Age 0.0272 0.1410 0.6944

Sex

Male 0.0000 0.0000 0.3846

Female 0.0000 0.0000 0.3846

Karnofsky Performance Status

30-40% 0.0128 0.0128 0.5494

50-60% 0.0128 0.0128 0.7959

70-80% 0.0513 0.0513 0.7692

90-100% 0.0256 0.0256 0.5071

Histological Grade

G1 0.0000 0.0000 0.0256

G2 0.0641 0.0641 0.9644

G3 0.0641 0.0641 1.1029

G4 0.0000 0.0000 0.1282

TNM-Classification

T1a 0.0385 0.0385 0.4619

T1b 0.0128 0.0128 0.6041

T2 0.0513 0.0513 0.3591

T2a 0.0000 0.0000 0.2051

Percent Balance Improvement:

Std. Mean Diff. Var. Ratio eCDF Mean eCDF Max

distance 79.9 60.2 92.9 56.0

Age 6.7 -9009.8 75.9 40.3

Sex

Male 100.0 . 100.0 100.0

Female 100.0 . 100.0 100.0

Karnofsky Performance Status

30-40% 76.1 . 76.1 76.1

50-60% 88.8 . 88.8 88.8

70-80% 31.5 . 31.5 31.5

90-100% 72.6 . 72.6 72.6

Histological Grade

G1 100.0 . 100.0 100.0

G2 -3542.9 . -3542.9 -3542.9

G3 -844.4 . -844.4 -844.4

G4 100.0 . 100.0 100.0

TNM-Classification

T1a 65.5 . 65.5 65.5

T1b 87.0 . 87.0 87.0

T2 72.0 . 72.0 72.0

T2a 100.0 . 100.0 100.0

Sample Sizes:

Control Treated

All 306 78

Matched 78 78

Unmatched 228 0

Discarded 0 0

Table S1: numeric results of propensity score matching

Figure S1: Histograms of propensity scores before and after matching. The histograms before matching on the left differ to a great degree. The histograms after matching on the right are very similar somehow. In sum, both the numerical and visual data show that the matching was successful.

| **Characteristic** | **N** | **HR***^1^* | **95% CI***^1^* | **p-value** |
| --- | --- | --- | --- | --- |
| Sex | 76 |  |  | 0.2 |
| female |  | — | — |  |
| male |  | 1.60 | 0.75, 3.42 |  |
| Patient Age | 76 | 1.01 | 0.98, 1.05 | 0.5 |
| Histological Grade | 42 |  |  | 0.3 |
| G2 |  | — | — |  |
| G3 |  | 1.43 | 0.47, 4.40 |  |
| G4 |  | 3.12 | 0.73, 13.2 |  |
| TNM-Classification | 76 |  |  | 0.9 |
| 1a |  | — | — |  |
| 1b |  | 1.06 | 0.54, 2.07 |  |
| Treated with SBRT | 76 | 1.12 | 0.57, 2.19 | 0.7 |
| Histological Grading available | 76 | 0.75 | 0.39, 1.44 | 0.4 |
| *^1^* HR = Hazard Ratio, CI = Confidence Interval | | | | |

Table S2: Univariate cox regression model of propensity matched data for T1-staged patients.
